# Insights on Late-Stage COVID-19 Pandemic Recovery From a 21-Country Online Survey

**DOI:** 10.3389/ijph.2025.1607601

**Published:** 2025-03-28

**Authors:** Louisa Ewald, John Bellettiere, Tamer H. Farag, Kristina M. Lee, Sidhartha Palani, Emma Castro, Amanda Deen, Catherine W. Gillespie, Bethany M. Huntley, Alison Tracy, Ana-Carolina Haensch, Frauke Kreuter, Wiebke Weber, Stefan Zins, Wichada La Motte-Kerr, Yao Li, Kathleen Stewart, Emmanuela Gakidou, Ali H. Mokdad

**Affiliations:** ^1^ Institute for Health Metrics and Evaluation, University of Washington, Seattle, WA, United States; ^2^ Department of Research, Meta, Redwood City, CA, United States; ^3^ Ludwig-Maximilian Universitat, Munich, Germany; ^4^ Department of Geographical Sciences, University of Maryland, College Park, MD, United States; ^5^ Institute for Employment Research of the German Federal Employment Agency, Nuremberg, Germany; ^6^ Department of Health Metrics Sciences, University of Washington, Seattle, WA, United States

**Keywords:** survey methodology, pandemic recovery, health care access, COVID-19 pandemic, vaccine confidence

## Abstract

**Objectives:**

The widespread impact of the COVID-19 pandemic on health systems, economies, and societies globally requires comprehensive data to guide effective recovery efforts. Online surveys have become crucial for rapid and extensive data collection. The Pandemic Response Survey (PRS), utilizing the Facebook Active User Base (FAUB), assessed the pandemic’s population-level impacts across 21 countries, gathering information on healthcare, vaccine confidence, trust, and economic and educational indicators.

**Methods:**

Conducted from March to May 2023, the PRS, translated into 15 languages, used the FAUB for gender-stratified random sampling of adults 18 years and older. The survey collected responses from 621,000 individuals, achieving a completion rate of 43%. Non-response and inverse propensity score weights were applied to calibrate the data to known demographic totals, enhancing the survey’s generalizability.

**Results:**

The PRS findings reveal disparities in life satisfaction, food security, delayed healthcare, vaccine confidence, and trust across countries. Life satisfaction was reported as high by 70%–80% of respondents in Egypt, Nigeria, Colombia, and Mexico, while only 20%–30% of respondents in Indonesia, Turkiye, and Viet Nam reported the same. Approximately 50% of respondents in Nigeria, South Africa, and Colombia experienced food insecurity, in contrast to less than 10% in Italy, Japan, and Germany. In Germany, 44% of respondents expressed high vaccine confidence compared to 10.6% in South Africa. Over half of respondents in Indonesia (52.4%) reported that their child was up to date on routine immunisations.

**Conclusion:**

The PRS demonstrates the effectiveness of online surveys in capturing actionable data during a global health crisis. The findings underscore the importance of targeted interventions and policy decisions to address the multifaceted challenges of pandemic recovery. Collaborative efforts in data collection and knowledge sharing between nations with shared profiles may foster more effective strategies.

## Introduction

The COVID-19 pandemic disrupted healthcare and brought sweeping changes to global health systems, economies, and societies [1–3]. Evidence indicates that healthcare delivery faced unprecedented challenges, with many non-COVID-related services being delayed or inaccessible, leading to widespread concerns over unmet health needs and the exacerbation of existing conditions [4–7]. Vaccine confidence has emerged as a critical issue, with misinformation and vaccine hesitancy posing significant barriers to immunization efforts against COVID-19, consequently affecting public health responses and recovery strategies [8]. Moreover, the pandemic has heightened food insecurity globally, with economic downturns and disruptions in food supply chains putting vulnerable populations at increased risk [9]. Additionally, routine childhood vaccination programs have experienced setbacks, risking the resurgence of vaccine-preventable diseases [10]. These impacts underscore the necessity for comprehensive data to address the multifaceted challenges posed by the pandemic and to inform effective recovery and preparedness measures for future health emergencies.

In the wake of such upheaval, gathering precise and comprehensive data on disruptions to health services becomes paramount for informed policy making. Such information is vital to implement effective recovery strategies and to prepare for future health crises with data-driven insights [3, 11].

As traditional data collection methods, including in-person or telephone interviews, face challenges in scope and timeliness, online surveys offer a promising alternative. Online surveys have become more prevalent as tools for health surveillance and policy research and are increasingly useful in the post-pandemic landscape. These methods are not only less expensive but also capable of rapidly engaging with large-scale populations and overcoming the limitations of traditional surveys, such as lower response rates and higher costs.

Throughout the pandemic, online surveys have played a pivotal role in gathering real-time data on infection rates, public behavior, and the effectiveness of health measures [5, 6, 12]. Large online surveys, such as the COVID-19 Trends and Impact Survey, which collected over 100 million responses across 114 countries, have demonstrated the capacity to provide valuable public health indicators [5, 6, 13]. The immediate flow of information enables health authorities and governments to understand needs and deploy resources accordingly.

The Pandemic Response Survey (PRS) builds upon the impact of online surveys in the post-pandemic world. Conducted from March to May 2023, the PRS spanned 21 countries, harnessing the collaborative efforts of the Institute for Health Metrics and Evaluation (IHME), LMU Munich (LMU), University of Maryland (UMD), and Meta. The primary objective of this extensive, cross-sectional internet-based survey was to generate a comprehensive understanding of the societal impacts of the COVID-19 pandemic, particularly in areas such as the economy, education, and health. The PRS stands as a response to the critical need for prevention and healthcare delivery, offering insights that are essential to shaping the future of global health policy. It represents a unique endeavor, not only due to its scale but also because of its employment of sampling and weighting procedures designed to mitigate non-response bias and coverage errors, enhancing the relevance and applicability of its findings.

## Methods

The main objective of this cross-sectional survey was to understand the wide-ranging impacts of the COVID-19 pandemic on societal and economic dimensions across 21 countries. Recognizing the pandemic’s multifaceted effects—beyond the direct health implications of the COVID-19 infection—this study aimed to capture a broad spectrum of experiences, including economic hardships, changes in social behaviors, and shifts in public attitudes towards health measures and governance.

### Sampling and Data Collection

Conducted between March and May 2023, the PRS harnessed the expansive reach of the Facebook Active User Base (FAUB) to sample a geographically and demographically diverse cohort from 21 strategically selected countries. The target population included active Facebook users aged 18 and over in the 21 selected countries. Countries were selected based on region, population, existing healthcare systems, and Facebook availability and usage [14–18].

The FAUB was divided into strata based on gender to ensure a balanced coverage of genders in the final sample. Within each gender strata, simple random samples were drawn, with the objective of obtaining an equivalent number of responses from individuals identifying as female and those identifying as non-female.

Respondents were invited via Facebook and redirected to the Qualtrics platform for survey completion. Participants were not able to take the survey twice or send the link to others. All participants provided informed consent prior to taking the survey. Meta did not have access to the data [19].

The questionnaire, translated into 15 languages, was crafted to facilitate comparability across different contexts. The selection of topics and questions was informed by benchmark surveys that addressed similar themes or were conducted in comparable populations or locations, allowing for the contextualization of the PRS data within the existing body of public health research [5, 6, 20, 21].

The computation of the survey weights consisted of two steps. First, response propensities were estimated for all persons who were invited to participate in the survey. Second, the inverse of the estimated response propensities were calibrated to known population totals of age, gender, and education categories and the regional distribution of the population [22, 23].

All materials and procedures for this study were reviewed and approved by the University of Washington Institutional Review Board (STUDY00016693). More detailed information on the methodology of the PRS, along with the full questionnaires and datasets, can be found in the Supplementary Appendix as well as through GESIS [24].

### Analysis

We calculated response rates as either completing the step-1 (answering country, region, age, education) and total completion as having made it to the last question.

Additionally, we compared the demographics of our sample to the known population statistics to evaluate the effectiveness of our sampling methodology. We calculated the proportion of survey respondents in each country-age-gender category to the corresponding category in the population estimates used for weighting.

We then looked at weighted descriptive results of several key indicators and conducted an in-depth Principal Component Analysis (PCA) to explore commonalities and differences across countries in terms of key indicators and generated a biplot to visualize the relationships both between the countries based on their scores on PC1 and PC2 and between the variables and the principal components. The vectors indicate the direction and magnitude of each variable’s influence on the principal components.

## Results

### Data Collection and Demographic Distribution

The PRS collected data from over 621,000 respondents across 21 countries, with respondents spending an average of 13.7 min to complete the questionnaire. The overall step-1-completion rate for all participants was 66.8%, and the total completion rate was 43.0%. Detailed completion rates for each country are available in the Supplementary Appendix.

The demographic distribution of our unweighted sample is detailed in Table 1, providing an overview of the age and gender composition of respondents by country as well as completion rates.

**TABLE 1:** Unweighted survey demographics by country. Includes counts and percentages of each demographic category by country. Data are from the Pandemic Recovery Survey, 2023 across 21 countries.

Country	Age (%)			Education (%)			Gender (%)			
	18–29 years	30–49 years	50+ years	Primary school or less	Secondary school	College or more	Female	Male	Prefer not to answer or non-binary
Argentina	3,348 (26.3)	5,775 (45.4)	3,593 (28.3)	3,120 (24.5)	6,517 (51.3)	3,079 (24.2)	5,948 (46.8)	6,570 (51.7)	198 (1.6)
Brazil	5,098 (23.1)	11,123 (50.3)	5,894 (26.7)	8,165 (36.9)	8,143 (36.8)	5,807 (26.3)	10,584 (47.9)	11,391 (51.5)	140 (0.6)
Chile	2042 (17.0)	5,660 (47.2)	4,299 (35.8)	1,604 (13.4)	5,897 (49.1)	4,500 (37.5)	6,045 (50.4)	5,789 (48.2)	167 (1.4)
Colombia	7,279 (40.6)	7,848 (43.7)	2,815 (15.7)	2,362 (13.2)	9,618 (53.6)	5,962 (33.2)	8,705 (48.5)	9,021 (50.3)	216 (1.2)
Egypt	14,351 (46.6)	13,363 (43.4)	3,064 (10.0)	1,238 (4.0)	8,914 (29.0)	20,626 (67.0)	12,417 (40.3)	17,943 (58.3)	418 (1.4)
Germany	962 (10.3)	4,284 (45.8)	4,108 (43.9)	481 (5.1)	6,401 (68.4)	2,472 (26.4)	5,050 (54.0)	4,195 (44.8)	109 (1.2)
India	17,004 (41.9)	20,126 (49.6)	3,483 (8.6)	3,298 (8.1)	7,002 (17.2)	30,313 (74.6)	18,575 (45.7)	21,804 (53.7)	234 (0.6)
Indonesia	12,496 (42.3)	14,335 (48.5)	2,740 (9.3)	3,793 (12.8)	17,639 (59.6)	8,139 (27.5)	13,557 (45.8)	15,654 (52.9)	360 (1.2)
Italy	1716 (9.8)	7,825 (44.7)	7,946 (45.4)	2,032 (11.6)	10,374 (59.3)	5,081 (29.1)	9,828 (56.2)	7,422 (42.4)	237 (1.4)
Japan	300 (3.0)	2,866 (28.7)	6,812 (68.3)	NR	4,729 (47.4)	5,178 (51.9)	4,270 (42.8)	5,580 (55.9)	128 (1.3)
Mexico	8,376 (36.5)	10,536 (45.9)	4,053 (17.6)	1,366 (5.9)	11,449 (49.9)	10,150 (44.2)	11,207 (48.8)	11,362 (49.5)	396 (1.7)
Nigeria	12,868 (47.0)	12,545 (45.9)	1945 (7.1)	706 (2.6)	8,421 (30.8)	18,231 (66.6)	9,527 (34.8)	17,708 (64.7)	123 (0.4)
Peru	6,090 (37.2)	7,001 (42.8)	3,269 (20.0)	915 (5.6)	8,234 (50.3)	7,211 (44.1)	7,898 (48.3)	8,309 (50.8)	153 (0.9)
Philippines	16,707 (42.9)	17,823 (45.7)	4,447 (11.4)	2,779 (7.1)	15,562 (39.9)	20,636 (52.9)	19,919 (51.1)	17,970 (46.1)	1,088 (2.8)
Poland	4,854 (29.9)	6,118 (37.7)	5,277 (32.5)	1,380 (8.5)	9,673 (59.5)	5,196 (32.0)	8,923 (54.9)	7,125 (43.8)	201 (1.2)
South Africa	9,603 (38.4)	12,100 (48.4)	3,279 (13.1)	1,256 (5.0)	14,770 (59.1)	8,956 (35.8)	13,105 (52.5)	11,585 (46.4)	292 (1.2)
Spain	1,044 (11.1)	4,199 (44.6)	4,165 (44.3)	2,065 (21.9)	3,884 (41.3)	3,459 (36.8)	5,197 (55.2)	4,110 (43.7)	101 (1.1)
Türkiye	1,597 (12.4)	7,049 (54.8)	4,213 (32.8)	2,346 (18.2)	6,209 (48.3)	4,304 (33.5)	5,279 (41.1)	7,491 (58.3)	NR
UK	1,093 (13.3)	3,291 (40.1)	3,829 (46.6)	342 (4.2)	3,128 (38.1)	4,743 (57.7)	4,197 (51.1)	3,913 (47.6)	103 (1.3)
United States of America	1,699 (16.0)	4,851 (45.6)	4,079 (38.4)	796 (7.5)	4,013 (37.8)	5,820 (54.8)	6,011 (56.6)	4,386 (41.3)	232 (2.2)
Viet Nam	14,204 (55.9)	9,177 (36.1)	2,019 (7.9)	1,634 (6.4)	12,478 (49.1)	11,288 (44.4)	11,492 (45.2)	13,344 (52.5)	564 (2.2)

NR, Not reportable due to insufficient sample size.

**FIGURE 1:**
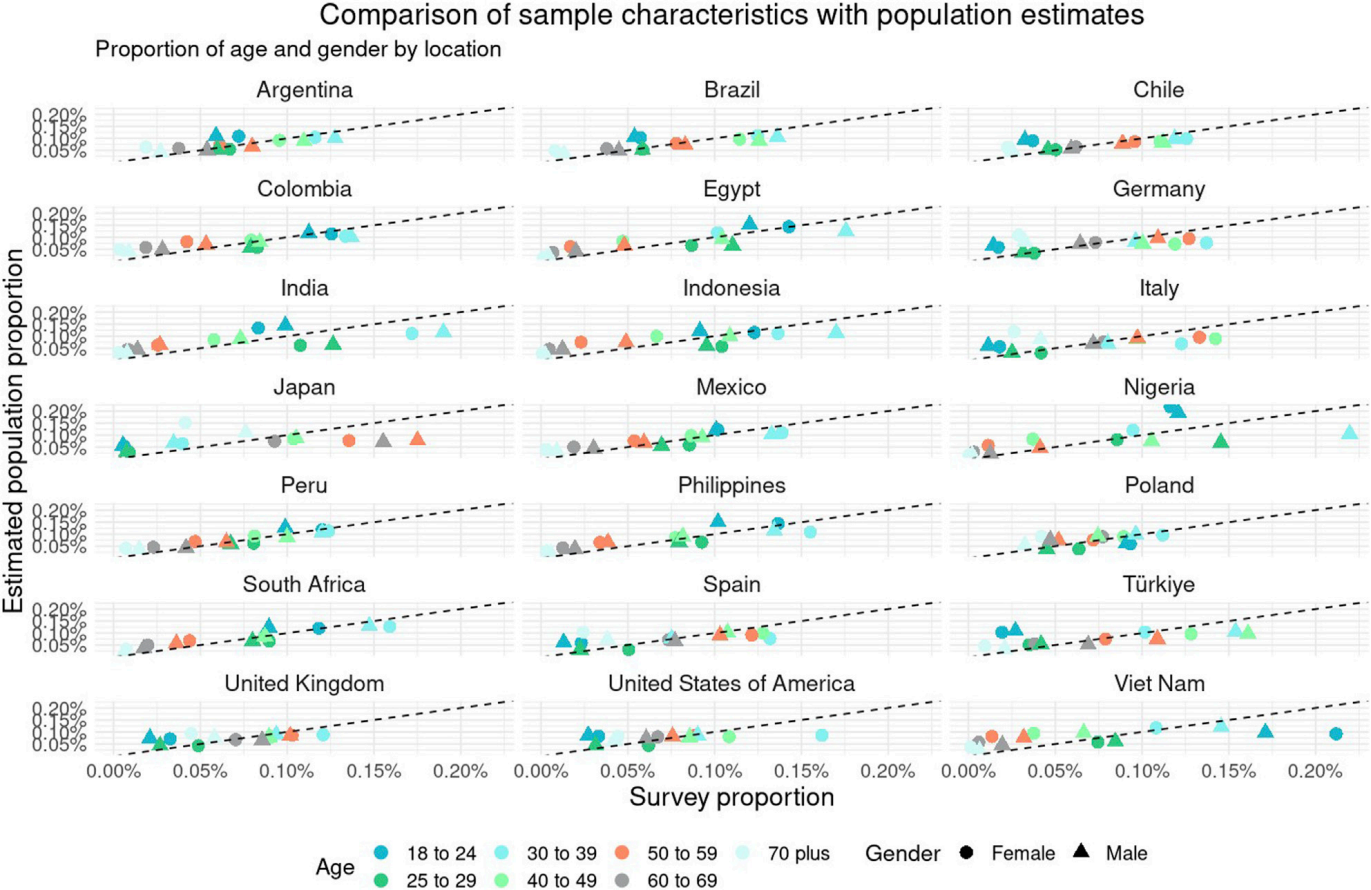
Compares the proportion of survey respondents in the Pandemic Recovery Survey with population estimates from the Global Burden of Disease across 21 countries. Survey proportions are stratified by country, age, and gender. Data are from the Pandemic Recovery Survey, 2023 across 21 countries.

Figure 1 details the comparison between unweighted survey characteristics and population estimates. Populations were categorized into location-age-gender buckets. Absolute differences between the survey respondent proportions and the estimated population proportions were minimal, with an average difference of 2.8% and a maximum difference of 11.9%.

### Findings From Key Indicators

**FIGURE 2:**
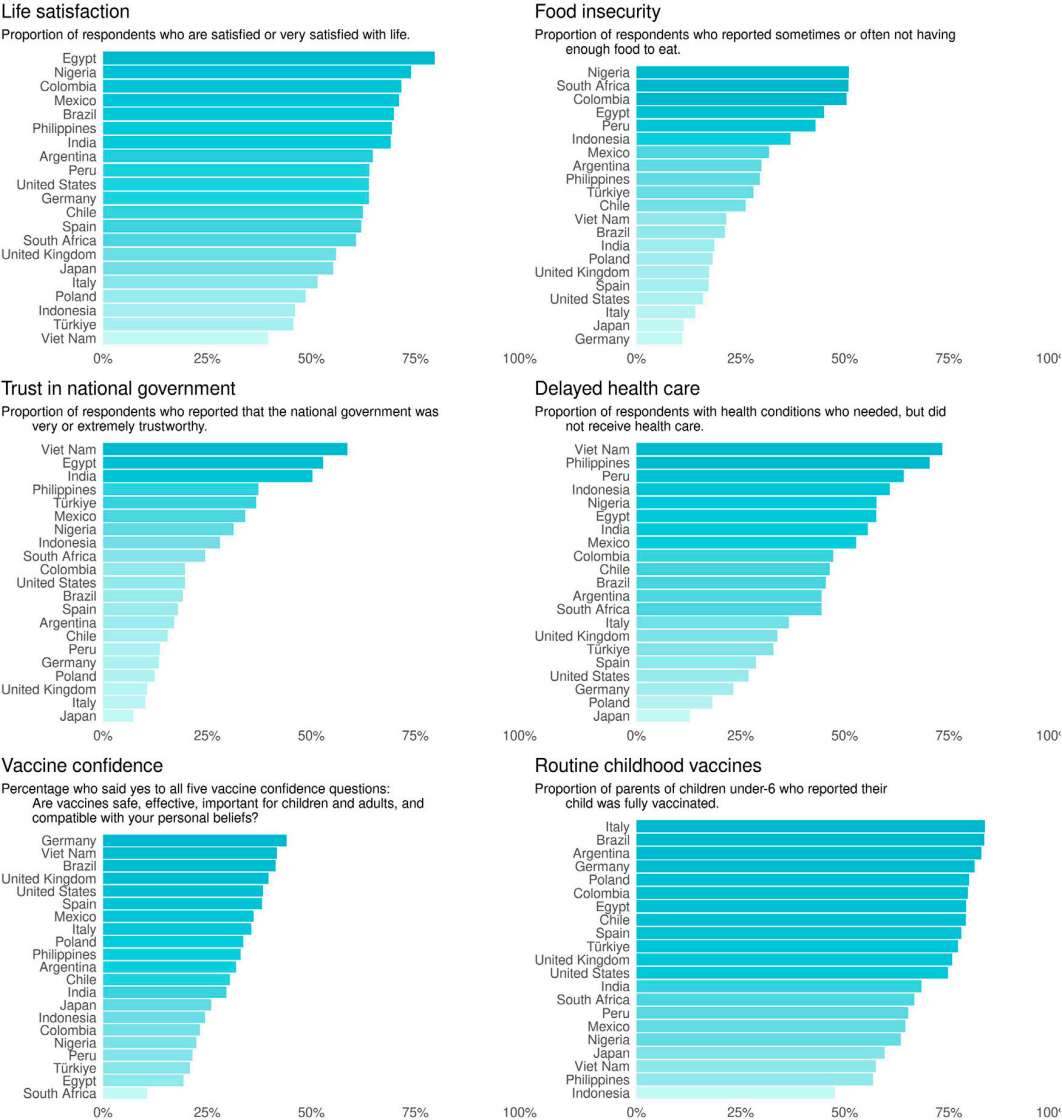
Presents a bar graph for six main indicators in the Pandemic Recovery Survey. Each graph shows the proportion of respondents from each country for each indicator including life satisfaction, trust in national government, vaccine confidence, food insecurity, delayed healthcare, and routine childhood vaccines. Data are from the Pandemic Recovery Survey, 2023 across 21 countries.

The survey revealed significant findings on various aspects of pandemic recovery. The proportion of respondents being satisfied or very satisfied with life, household expenses and food security, delayed healthcare, vaccine confidence, and the perceived decline in math skills of students are presented in Figure 2. These findings illustrate the wide variation in experiences and perceptions across different countries.

In an analysis of key indicators, we observed notable variations in public sentiment and experiences across the 21 countries surveyed. Life satisfaction levels varied widely, with respondents in Egypt, Nigeria, Colombia, and Mexico reporting the highest levels of contentment, while respondents in Indonesia, Türkiye, and Viet Nam showed considerably lower satisfaction. Food insecurity emerged as a significant concern, particularly for respondents in Nigeria, South Africa, and Colombia, where half reported a lack of sufficient food. Conversely, respondents in Italy, Japan, and Germany reported the lowest levels of food insecurity. The analysis also highlighted issues with delayed healthcare, especially pronounced in Viet Nam and the Philippines, where the majority of respondents with medical conditions experienced delays in receiving care. In terms of public trust in vaccines, there was a clear disparity between countries, with more than 40% of respondents in Germany, Viet Nam, and Brazil showing high confidence in vaccine safety, efficacy, and alignment with personal beliefs, whereas less than 20% of respondents in South Africa and Egypt showed the same level of confidence. Trust in the national government displayed large gaps between countries, with Viet Nam, Egypt, and India among the most trusting, and the UK, Italy, and Japan the least trusting of the national government. Finally, parents of children under 5 reported whether their child was fully vaccinated with routine childhood immunizations, and a majority of countries had more than 70% of respondents reporting full vaccination, however, less than half of respondents in Indonesia (47.6%) reported that their child was fully vaccinated. These high-level results from the combined plot provide a snapshot of the multifaceted challenges faced by different countries in the wake of COVID-19.

### Patterns of Country Similarities

The PCA identified distinct clusters of countries that share similar profiles with respect to life satisfaction, vaccine confidence, food security, trust in national government, and COVID-19 vaccination status. The first principal component (PC1) explained a significant proportion of the variance (47.5%), with food security showing the most substantial positive loading, followed by vaccine confidence and COVID-19 vaccination status. This suggests that PC1 captures an aspect of public health and welfare, with higher scores correlating with better food security, vaccine confidence, and vaccination rates. Conversely, PC1 had a slight negative relationship with life satisfaction, indicating a modest inverse correlation with life satisfaction.

The second principal component (PC2) accounted for the next largest variance (23.5%) and was most strongly positively associated with life satisfaction. On the other hand, a strong negative loading on trust in national government implies that higher PC2 scores may also reflect a diminished trust in national government.

Together, PC1 and PC2 cumulatively captured 71% of the total variance. The remaining components, PC3 through PC5, contributed to capturing smaller, yet meaningful, aspects of the variance, with strong negative loadings on life satisfaction and trust in national government for PC3, a positive relationship with COVID-19 vaccination status on PC4, as well as a positive relationship for food security on PC5.

Table 2 presents the loadings of 21 countries on the first two principal components derived from an analysis focusing on life satisfaction, vaccine confidence, food security, trust in national government, and COVID-19 vaccination status. PC1 is strongly associated with health-related metrics, while PC2 is linked to life satisfaction and trust in national government. Data are from the Pandemic Recovery Survey, 2023 across 21 countries.

**TABLE 2:** Principal Component Analysis: Country scores across five health, life satisfaction, and trust indicators. Data are from the Pandemic Recovery Survey, 2023 across 21 countries.

Country	PC1	PC2
Argentina	−0.043	0.429
Brazil	1.278	1.084
Chile	0.527	0.531
Colombia	−1.117	0.566
Egypt	−2.734	−0.535
Germany	1.298	0.903
India	0.191	−1.149
Indonesia	−0.675	−1.338
Italy	1.664	0.503
Japan	0.621	0.161
Mexico	0.821	0.602
Nigeria	−3.964	0.822
Peru	−0.187	0.454
Philippines	0.166	−0.231
Poland	0.474	0.245
South Africa	−2.876	−0.233
Spain	1.247	0.615
Türkiye	−0.193	−1.709
United Kingdom	1.426	0.696
United States	0.388	0.904
Viet Nam	1.688	−3.321

**FIGURE 3:**
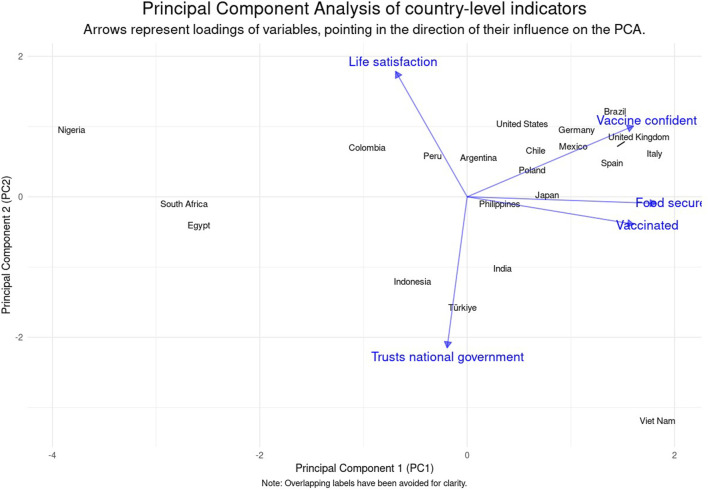
Displays the distribution of countries based on their scores on the first two principal components, illustrating the relationship between life satisfaction, vaccine confidence, food security, trust in national government, and COVID-19 vaccination status. The diagram identifies clusters of countries with similar health and governance profiles. Data are from the Pandemic Recovery Survey, 2023 across 21 countries.

Figure 3 illustrates clusters of countries that share similar profiles regarding the considered indicators. Countries with closer proximity on the plot are more alike in terms of the underlying variables. For instance, countries in the upper right quadrant (Brazil, Germany, UK, Spain, and Italy) typically exhibit high life satisfaction and vaccine confidence but may have lower trust in government. In contrast, those in the lower left quadrant (South Africa, Egypt, Indonesia, Türkiye) tend to have lower food security and vaccination rates, while having a higher trust in the national government.

## Discussion

The Pandemic Response Survey (PRS) has provided many valuable insights into the varied impacts of the COVID-19 pandemic across 21 countries. Utilizing the Facebook Active User Base as a novel sampling frame, the PRS successfully captured a broad demographic, illustrating the potential of online surveys to rapidly collect large-scale data in a global crisis setting at a low cost.

The data collected through the PRS is invaluable for guiding policy and can serve as a baseline for monitoring progress. This will provide insights for planning and implementing programs and policies aimed at addressing the challenges ahead.

Key findings include the large disparities in life satisfaction, food security, delayed healthcare, vaccine confidence, and trust in national government across countries. For instance, life satisfaction was reported as high by 70%–80% of respondents in Egypt, Nigeria, Colombia, and Mexico, in contrast to only 20%–30% of respondents in Indonesia, Türkiye, and Viet Nam. Respondents in Egypt showed some of the highest levels of trust in government with 52.8% responding that the national government is either trustworthy or very trustworthy. Although governmental trust in Egypt is among the highest in the Arab world, the rate of life satisfaction, 79.6%, is higher than other reports, possibly from the subset of the population most likely to be active on Facebook and answer surveys [25–27].

The survey highlighted food insecurity as a pressing concern in several countries, specifically in Nigeria, South Africa, Colombia, Egypt, and Peru. These findings resonate with reports from the United Nations World Food Programme that pandemic-related food insecurity have been amplified by conflict, climate, and food access [9].

Another indicator that revealed vast disparities was in delayed healthcare, with some countries reporting high amounts of respondents who experienced delayed healthcare. Specifically, 73.3% of respondents in Viet Nam, 70.3% in the Philippines, and 64.1% in Peru reported needing healthcare but not receiving. Other population-based surveys during this time period similarly reported overall low healthcare quality, specifically in low- and middle-income countries (LMICs) and populations with high health needs, low education, or low income [28].

The analysis also revealed disparities in vaccine confidence, with Germany, Viet Nam, and Brazil showing more than 40% of respondents expressing high vaccine confidence, compared to just 10.6% in South Africa. Even in Germany, where respondents reported the highest level of vaccine confidence, barriers to vaccination still exist including trust in institutions and healthcare professionals [29]. Respondents in Egypt showed one of the lowest rates of vaccine confidence (19.3%) other than South Africa (10.6%), along with less than a quarter of respondents expressing confidence in Türkiye, Peru, Nigeria, Colombia, and Indonesia. These findings are comparable to previous studies examining vaccine confidence and finding overall low rates in Egypt, Indonesia, the Philippines, and several other countries [10, 30, 31].

The PRS findings underscore the necessity for a nuanced, multifaceted approach to pandemic recovery that considers the socio-economic, cultural, and political contexts of each country. Collaborative efforts in data collection, as demonstrated by the PRS, are vital for understanding and addressing the global impacts of health crises. Our findings suggest that policies should prioritize food security, healthcare access, and vaccine confidence building, tailored to the specific needs of each population.

The survey also highlights the potential of online platforms for rapid, large-scale data collection during global health emergencies. As we move forward, leveraging technology for health surveillance and policy research will be increasingly important. However, efforts must be made to ensure these methods are inclusive and representative of all demographic groups.

### Limitations

Despite its strengths, the PRS has limitations, including potential under-representation of certain demographic groups such as older adults or those without internet access. Efforts to mitigate non-response bias through advanced statistical techniques may not fully account for differences between respondents and non-respondents. The result of this limitation leads to uncertainty in the results due to certain populations that are underrepresented, specifically underserved and hard to reach populations. The survey data are also fully self-report and susceptible to social desirability bias, recall bias, and other risks associated with self-report data. The demographic makeup of the FAUB may also distort differences between countries. Additionally, the country-level findings are not age-standardized, which may influence the results. Since certain age groups may respond in systematically different ways, future research analyzing results for all ages should account for age differences.

### Conclusion

The insights gained from the PRS, particularly regarding disparities in food security, delayed healthcare, and vaccine confidence, have important implications for global health policy and post-pandemic recovery efforts. The survey’s findings can inform strategies to improve vaccine uptake, strengthen public trust in health interventions, and address educational disruptions.

The PRS exemplifies the role of online surveys in capturing complex realities of a global health emergency. Moving forward, it is critical to build upon the lessons learned from the PRS, continuing to enhance the quality and coverage of online survey data and its application to global health policy and practice.

## Data Availability

Deidentified participant-level microdata are available for download on GESIS at https://doi.org/10.7802/2631.

## References

[1] Haileamlak A. The Impact of COVID-19 on Health and Health Systems. Ethiop J Health Sci (2021) 31(6):1073–4. 10.4314/ejhs.v31i6.1 35392335 PMC8968362

[2] WHO Team. Second Round of the National Pulse Survey on Continuity of Essential Health Services during the COVID-19 Pandemic. In: Report No.: WHO/2019-nCoV/EHS_continuity/survey/2021.1 (2021). Available online at: https://www.who.int/publications/i/item/WHO-2019-nCoV-EHS-continuity-survey-2021.1. (Accessed May 8, 2024).

[3] Bashier H Ikram A Khan MA Baig M Al Gunaid M Al Nsour M The Anticipated Future of Public Health Services Post COVID-19: Viewpoint. JMIR Public Health Surveill (2021) 7(6):e26267. 10.2196/26267 33592576 PMC8216329

[4] Papautsky EL Hamlish T. Patient-Reported Treatment Delays in Breast Cancer Care during the COVID-19 Pandemic. Breast Cancer Res Treat (2020) 184(1):249–54. 10.1007/s10549-020-05828-7 32772225 PMC7415197

[5] Astley CM Tuli G Mc Cord KA Cohn EL Rader B Varrelman TJ Global Monitoring of the Impact of the COVID-19 Pandemic through Online Surveys Sampled from the Facebook User Base. Proc Natl Acad Sci USA (2021) 118(51):e2111455118. 10.1073/pnas.2111455118 34903657 PMC8713788

[6] Salomon JA Reinhart A Bilinski A Chua EJ La Motte-Kerr W Rönn MM The US COVID-19 Trends and Impact Survey: Continuous Real-Time Measurement of COVID-19 Symptoms, Risks, Protective Behaviors, Testing, and Vaccination. Proc Natl Acad Sci USA (2021) 118(51):e2111454118. 10.1073/pnas.2111454118 34903656 PMC8713763

[7] Kruk ME Kapoor NR Lewis TP Arsenault C Boutsikari EC Breda J Population Confidence in the Health System in 15 Countries: Results from the First Round of the People’s Voice Survey. The Lancet Glob Health (2024) 12(1):e100–11. 10.1016/S2214-109X(23)00499-0 38096882 PMC10716625

[8] Troiano G Nardi A. Vaccine Hesitancy in the Era of COVID-19. Public Health (2021) 194:245–51. 10.1016/j.puhe.2021.02.025 33965796 PMC7931735

[9] World Food Programme. A Global Food Crisis (WFP Annual Review) (2023). Available online at: https://www.wfp.org/global-hunger-crisis. (Accessed May 8, 2024).

[10] Saied SM Saied EM Kabbash IA. Vaccine Hesitancy: Beliefs and Barriers Associated with COVID-19 Vaccination Among Egyptian Medical Students. J Med Virol (2021) 93(7):4280–91. 10.1002/jmv.26910 33644891 PMC8013865

[11] Stoto MA Kraemer JD Piltch-Loeb R. Better Metrics to Guide Public Health Policy: Lessons Learned from COVID-19 for Data Systems Improvement. Harv Data Sci Rev (2023) 5(1). 10.1162/99608f92.3e516c04

[12] Lazarus JV Ratzan S Palayew A Billari FC Binagwaho A Kimball S COVID-SCORE: A Global Survey to Assess Public Perceptions of Government Responses to COVID-19 (COVID-SCORE-10). PLoS ONE 15 (10), e0240011 (2020). 10.1371/journal.pone.0240011 33022023 PMC7538106

[13] Fan J Li Y Stewart K Kommareddy AR Andres Garcia The University of Maryland Social Data Science Center Global COVID-19 Trends and Impact Survey. In: Partnership with Facebook (2020). Available online at: https://covidmap.umd.edu/api.html. (Accessed May 8, 2024).

[14] Wang H Abbas KM Abbasifard M Abbasi-Kangevari M Abbastabar H Abd-Allah F Global Age-Sex-Specific Fertility, Mortality, Healthy Life Expectancy (HALE), and Population Estimates in 204 Countries and Territories, 1950–2019: A Comprehensive Demographic Analysis for the Global Burden of Disease Study 2019. The Lancet (2020) 396(10258):1160–203. 10.1016/S0140-6736(20)30977-6 PMC7566045 33069325

[15] Federal Statistical Office of Germany (DeStatis). GENESIS- Online Database (2025). Available online at: https://www.destatis.de/EN/Home/_node.html. (Accessed May 8, 2024).

[16] Office for the Coordination of Humanitarian Affairs (OCHA). Subnational Population. Humanitarian Data Exchange. Available online at: https://data.humdata.org/. (Accessed May 8, 2024).

[17] Japanese Government Statistics. Population Estimates (2024). Available online at: https://www.e-stat.go.jp/en. (Accessed May 8, 2024).

[18] Instituto Nacional de Estadistica. Demography and Population. Available online at: https://www.ine.es/en/index.htm. (Accessed May 8, 2024).

[19] Qualtrics. Qualtrics. Provo, UT, USA: Qualtrics (2005). Available online at: https://www.qualtrics.com (Accessed May 8, 2024).

[20] Larson HJ Schulz WS Tucker JD Smith DMD. Measuring Vaccine Confidence: Introducing a Global Vaccine Confidence Index. Plos Curr (2015) 7. 10.1371/currents.outbreaks.ce0f6177bc97332602a8e3fe7d7f7cc4 PMC4353663 25789200

[21] Gates B Gates M. Goalkeepers Report. In: Bill and Melinda Gates Foundation (2020). Available online at: https://www.gatesfoundation.org/goalkeepers/report/2020-report/#GlobalPerspective. (Accessed May 8, 2024).

[22] Rosenbaum PR Rubin DB. The Central Role of the Propensity Score in Observational Studies for Causal Effects. Biometrika (1983) 70(1):41–55. 10.2307/2335942

[23] Sarig T Galili T Eilat R. Balance -- a Python Package for Balancing Biased Data Samples (2023). Available online at: https://arxiv.org/abs/2307.06024 (Accessed March 13, 2024).

[24] Haensch A Kreuter F La Motte-Kerr W Stewart K Weber W Zins S Pandemic Recovery Survey. (College Park, MD: The University of Maryland). Available online at: https://healthsurveys.umd.edu. (Accessed May 8, 2024).

[25] Kayyali AW. The Arab World’s Trust in Government and the Perils of Generalization. (Ann Arbor, MI: University of Michigan) (2020). Available online at: https://www.arabbarometer.org/2020/06/the-arab-worlds-trust-in-government-and-the-perils-of-generalization/#:∼:text=According%20to%20Arab%20Barometer%20Wave,just%2010%20percent%20in%20Libya. (Accessed May 8, 2024).

[26] El-Monshed A Loutfy A Saad M Ali A El-Gilany AH Soliman MA Satisfaction with Life and Psychological Distress during the COVID-19 Pandemic: An Egyptian Online Cross-Sectional Study. Afr J Prim Health Care Fam Med (2022) 14(1):e1–e6. 10.4102/phcfm.v14i1.2896 PMC8831996 35144450

[27] Department of Statistics and Censuses (Egypt). Central Agency for Public Mobilization and Statistics (CAPMAS). Available online at: http://capmas.gov.eg/. (Accessed May 8, 2024).

[28] Lewis TP Kassa M Kapoor NR Arsenault C Bazua-Lobato R Dayalu R User-Reported Quality of Care: Findings from the First Round of the People’s Voice Survey in 14 Countries. The Lancet Glob Health (2024) 12(1):e112–22. 10.1016/S2214-109X(23)00495-3 38096883 PMC10716624

[29] Sterl S Stelzmann D Luettschwager N Gerhold L. COVID-19 Vaccination Status in Germany: Factors and Reasons for Not Being Vaccinated (Yet). Front Public Health (2023) 11:1070272. 10.3389/fpubh.2023.1070272 36860382 PMC9969553

[30] Omar DI Hani BM. Attitudes and Intentions towards COVID-19 Vaccines and Associated Factors Among Egyptian Adults. J Infect Public Health (2021) 14(10):1481–8. 10.1016/j.jiph.2021.06.019 34247946 PMC8253692

[31] De Figueiredo A Simas C Karafillakis E Paterson P Larson HJ. Mapping Global Trends in Vaccine Confidence and Investigating Barriers to Vaccine Uptake: A Large-Scale Retrospective Temporal Modelling Study. The Lancet (2020) 396(10255):898–908. 10.1016/S0140-6736(20)31558-0 PMC7607345 32919524

