# Supplementary material for: Insights on Late-Stage COVID-19 Pandemic Recovery From a 21-Country Online Survey
Source: Int J Public Health. 2025 Mar 28;70:1607601. doi: 10.3389/ijph.2025.1607601 (PMC11985331; doi:10.3389/ijph.2025.1607601)
Supplement: Supplementary file 1 [file DataSheet1.docx]

**Appendix**

**Table 1: Survey completion rates by country**

| **Country** | **Total number of respondents** | **Step1 completion rate** | **Total completion rate** |
| --- | --- | --- | --- |
| Argentina | 12,716 | 69.0% | 48.0% |
| Brazil | 22,115 | 72.2% | 50.6% |
| Chile | 12,001 | 68.7% | 48.4% |
| Colombia | 17,942 | 69.6% | 48.1% |
| Egypt | 30,778 | 74.8% | 40.4% |
| Germany | 9,354 | 68.0% | 52.2% |
| India | 40,613 | 75.0% | 33.6% |
| Indonesia | 29,571 | 74.9% | 46.3% |
| Italy | 17,487 | 67.3% | 51.7% |
| Japan | 9,978 | 61.5% | 51.7% |
| Mexico | 22,965 | 74.5% | 53.9% |
| Nigeria | 27,358 | 89.9% | 61.9% |
| Peru | 16,360 | 68.6% | 46.3% |
| Philippines | 38,977 | 96.4% | 62.7% |
| Poland | 16,249 | 61.5% | 41.6% |
| South Africa | 24,982 | 74.3% | 48.5% |
| Spain | 9,408 | 63.7% | 44.4% |
| Türkiye | 12,859 | 67.6% | 43.1% |
| UK | 8,213 | 58.3% | 44.8% |
| USA | 10,629 | 50.5% | 37.7% |
| Viet Nam | 25,400 | 66.1% | 31.3% |

Methodological details

*Country selection*

The Pandemic Response Survey (PRS) encompassed 21 countries, strategically chosen to provide a comprehensive cross-section of global populations. Selection criteria included population size, geographical diversity, internet penetration rates, Facebook usage statistics, and prior collaborations with the Global Burden of Disease study. These factors ensured a diverse and representative sample capable of capturing a wide range of pandemic-related experiences. Selected countries as shown in Figure 1 are Argentina, Brazil, Chile, Colombia, Egypt, Germany, India, Indonesia, Italy, Japan, Mexico, Nigeria, Peru, Philippines, Poland, South Africa, Spain, Turkiye, the United Kingdom, the United States, and Viet Nam.

**Figure 1. Number of survey responses by country**

*
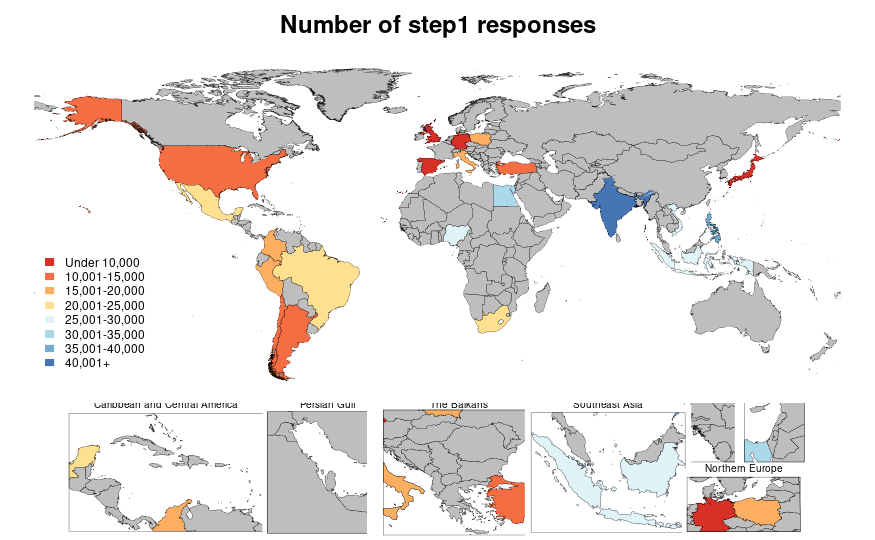
*

*Sampling methodology*

The PRS employed the Facebook Active User Base (FAUB) as a novel sampling frame, targeting individuals aged 18 and older in the selected countries. The FAUB facilitated access to a diverse sample, taking advantage of high internet and social media penetration rates globally. The FAUB was divided into strata based on gender to ensure a balanced coverage of genders in the final sample.

Within each gender strata, simple random samples were drawn, with the objective of obtaining an equivalent number of responses from individuals identifying as female and those identifying as non-female. This approach was carefully devised to mirror the gender distribution within the FAUB in each country, thereby minimizing sampling bias and enhancing the reliability of the survey findings.

Using general sample size guidelines, to estimate an outcome that affects approximately 50% of the population with 95% confidence and a margin of error of 5%, a minimum of 385 complete responses are needed for each subgroup-specific signal or desired prevalence estimate. Inflating this value by 15% to account for item non-response yields 443 completed surveys per age-, sex-, and location-specific subgroup of interest.

*Questionnaire design and implementation*

The questionnaire of the PRS was crafted to facilitate comparability across different contexts. The selection of topics and questions was informed by benchmark surveys that addressed similar themes or were conducted in comparable populations or locations, allowing for the contextualisation of the PRS data within the existing body of public health research.[^5,6,14,15^](https://www.zotero.org/google-docs/?2pio7v) The Institute for Health Metrics and Evaluation (IHME) spearheaded the questionnaire design process, incorporating insights from public health experts to refine the survey content while collaborating with Meta, UMD and LMU to ensure the questionnaire’s methodological rigor.

The PRS questionnaire, along with its invitations and instructions, was translated into 15 languages. Professional translators carried out the translation work, and native speakers performed subsequent reviews to maintain linguistic and contextual accuracy. These languages were chosen to encompass at least 95% of the population in the surveyed countries, maximizing the reach and inclusivity of the survey. Public health officials provided valuable feedback, helping to refine medical terminology and ensuring that translations accurately conveyed the intended meanings of survey questions.

Data were collected by UMD using Data collection time ranged from 16 March 2023  to 16 May 2023, with survey invitations sent to each country until our needed sample size was achieved. Dates of the last survey response recorded by country are presented in Table 1. All respondents provided informed consent. A data pipeline was created that pulled the response data from Qualtrics and computed several survey monitoring metrics relating to data quality and completion rates and attached the survey weights.

**Weighting methodology**

Survey non-response was adjusted for using inverse propensity score weighting (IPW).[^21^](https://www.zotero.org/google-docs/?hacqF7) We first computed the estimated response propensity using logistic regression with LASSO (Least Absolute Shrinkage and Selection Operator).[^6^](https://www.zotero.org/google-docs/?umvKgy) All users who received an invitation were included in this modeling. The dependent variable was whether or not a user 1) consented to the survey, 2) completed the first four survey questions about demographics, and 3) completed at least two survey questions. The covariates included in this model included age, gender, and country of residence reported by users on the Facebook app, and also other information about how sampled users interact with the Facebook app. Continuous covariates were categorized to fit the covariate distribution better by bucketing the data into deciles. Missing data were addressed by creating a separate category for missingness for each variable. The inverse of the response propensity was then scaled to the target population (ie, the total number of users sent a survey invitation). To control for the variation of weights, a bound of 1·5 was set for the factor of Kish’s design effect that measures the effect of using unequal weights on the variance of a weighted sample mean.[^22^](https://www.zotero.org/google-docs/?V5PVcl)

**Computation of the final survey weights**

We provided weights to enable inference to the target population (people being at least 18 years old) of each country in the survey. The weights are scaled to the size of the target population.

For the observations of each country, the inverse of the estimated response propensities were calibrated, using a linear calibration method, to the population totals of the following socio-demographic variables:

1. Cross-classification of gender and age
2. Cross-classification of gender and education
3. Cross-classification of age and education
4. Region

with the following categories:

1. Age: 18–24; 25–29; 30–39; 40–49; 50–59; 60–69; 70+
2. Gender: Female; non-female
3. Education: Highest level of completed education: none; primary; secondary; tertiary
4. Region: Location of residence by subnational administrative level 1 regions

The calibration was done for two sets of the sample, for the step-1-complete responses and the partial complete responses. Step 1 is achieved when respondents consent to take part in the survey, confirm that they are 18 years or older and state the country and region they live in, their age group, gender, and education level, in addition to answering two other questions. Partial complete is achieved when respondents in addition to the step-1 requirements answer any two of the questions 36, 37, and 38.

For all countries, the step-1-complete weights were trimmed to be between 1 and 100,000. For partial complete responses, only a lower bound of 1 was set. The trimming of the step-1-complete weights results in a substantially lower coefficient of variation (CV) of the weights for some countries, which displays the relative change between the CV of the trimmed and the CV of the untrimmed weights.

Using the provided survey weights should increase the chances that the results more accurately reflect the characteristics of the target population in each country, but it is not certain that they ensure an approximately unbiased estimation.

Also the survey weights do not perfectly replicate the population totals used in the calibration, as there is a trade-off between bounding the weights and achieving a better fit to the population totals. It is possible that non-respondents differ from respondents conditional on the variables used in the estimation of the response propensities and calibration and/or that Facebook users differ from the target population even if conditioned on the variables that are used in the calibration.

**Population totals for weighting**

Population totals for the categories of variables age, gender, and education come from the 2019 Global Burden of Disease study.[^23^](https://www.zotero.org/google-docs/?dmvWpH)

The population totals for the subnational administrative level 1 regions were obtained from different sources. For Brazil, India, Indonesia, Italy, Mexico, Poland, South Africa, the UK, and the USA, the data were also based on estimates provided by IHME.

For Argentina, Chile, Colombia, Nigeria, Peru, the Philippines,Türkiye, and Viet Nam, the percentages of the total population by  subnational administrative level 1 region were obtained from datasets provided by the Humanitarian Data Exchange.[^24^](https://www.zotero.org/google-docs/?yMULKn) For Egypt, data were obtained from the Central Agency for Public Mobilization And Statistics (CAPMAS);[^25^](https://www.zotero.org/google-docs/?eOEpDn) for Germany, from the Federal Statistical Offices (DeStatis);[^26^](https://www.zotero.org/google-docs/?xwH8gt) for Japan, from e-stat, a portal site for Japanese Government Statistics;[^27^](https://www.zotero.org/google-docs/?ZXUTi2) and for Spain, from the Spanish Statistical Office.[^28^](https://www.zotero.org/google-docs/?bVeoOB) The percentages per subnational administrative level 1 were multiplied by the respective overall population total estimated by IHME.

The survey data underwent processing both in Qualtrics and the data pipeline to ensure the validity and reliability of the collected data. The checks were carried out to ensure the data were of high quality and errors or inconsistencies were dealt with appropriately. The data were examined for duplicates, illogical codes, and other data quality issues such as straight-lining, skip and “don’t know” rates, and illogical time spent on survey as a whole.
